# ZC3H13 Inhibits the Progression of Hepatocellular Carcinoma through m^6^A-PKM2-Mediated Glycolysis and Enhances Chemosensitivity

**DOI:** 10.1155/2021/1328444

**Published:** 2021-12-30

**Authors:** Qibo Wang, Haichuan Xie, Hao Peng, Jianjian Yan, Limin Han, Gang Ye

**Affiliations:** ^1^Department of Hepatobiliary Surgery, Pingxiang People's Hospital of Southern Medical University, Pingxiang, Jiangxi 337055, China; ^2^Department of Hepatobiliary Surgery, People's Hospital of Changshou Chongqing, Chongqing 401220, China; ^3^Department of Endocrinology, People's Hospital of Changshou Chongqing, Chongqing 401220, China

## Abstract

**Objective:**

N^6^-Methyladenosine (m^6^A) is the most prevalent RNA epigenetic modulation in eukaryotic cells, which serves a critical role in diverse physiological processes. Emerging evidences indicate the prognostic significance of m^6^A regulator ZC3H13 in hepatocellular carcinoma (HCC). Herein, this study was conducted for revealing biological functions and mechanisms of ZC3H13 in HCC.

**Methods:**

Expression of ZC3H13 was examined in collected HCC and normal tissues, and its prognostic significance was investigated in a public database. Gain/loss of functional assays were presented for defining the roles of ZC3H13 in HCC progression. The specific interactions of ZC3H13 with PKM2 were validated in HCC cells via mRNA stability, RNA immunoprecipitation, and luciferase reporter and MeRIP‐qPCR assays. Moreover, rescue experiments were carried out for uncovering the mechanisms.

**Results:**

ZC3H13 expression was downregulated in HCC, and its loss was in relation to dismal survival outcomes. Functionally, overexpressed ZC3H13 suppressed proliferation, migration, and invasion and elevated apoptotic levels of HCC cells. Moreover, ZC3H13 overexpression sensitized to cisplatin and weakened metabolism reprogramming of HCC cells. Mechanically, ZC3H13-induced m^6^A modified patterns substantially abolished PKM2 mRNA stability. ZC3H13 facilitated malignant behaviors of HCC cells through PKM2-dependent glycolytic signaling.

**Conclusion:**

Collectively, ZC3H13 suppressed the progression of HCC through m^6^A-PKM2-mediated glycolysis and sensitized HCC cells to cisplatin, which offered a fresh insight into HCC therapy.

## 1. Introduction

Liver carcinoma represents the most frequent fatal malignant disease across the globe [1]. Among all liver carcinoma patients, hepatocellular carcinoma (HCC) occupies over 90% [2]. Patients' survival outcomes are dismal. Merely 5%–15% of patients benefit from radical resection, only for those in the earlier stages [3]. Therapeutic strategies for advanced-stage patients contain transarterial chemoembolization (TACE) as well as oral sorafenib [4]. Nevertheless, <33% of patients do not respond to this therapy as well as develop marked chemotherapy resistance within 6 months from starting therapeutic intervention [5]. Moreover, long-term usage of chemotherapeutic agents causes toxic response as well as chemotherapeutic inefficiency [6]. Therefore, neither TACE nor chemotherapeutic agents can remarkedly improve the outcome of liver cancer. In-depth exploration is required for finding a better way to treat liver cancer.

N^6^-methyladenosine (m^6^A) is the most prevalent form of internal mRNA modification [7]. m^6^A modification has been proposed as the most frequent chemical modified form in eukaryotic mRNAs [8], which is of importance for controlling diverse cellular and biological events like RNA stability, translation, and splicing [6]. As estimated, about 0.1%–0.4% of adenosine in mRNAs may be modified via m^6^A, with a mean of 2-3 m^6^A modified sites per transcript [9]. m^6^A modification patterns are dominated through methyltransferase complex (“writer”), demethylase (“eraser”), and RNA-binding protein (“reader”) [10]. Emerging evidences highlight the significance of deregulation of m^6^A modification in liver carcinogenesis [9]. Through comprehensive analyses of m^6^A regulators in TCGA-HCC project, Liu et al. proposed that METTL3, YTHDF2, and ZC3H13 acted as independent prognostic indicators of HCC outcomes [11]. METTL3 expression exhibited a frequent upregulation in HCC and promoted HCC development via YTHDF2-dependent posttranscriptional silence of SOCS2 [12]. Another study proposed the mechanisms of SUMOylated METTL3-mediated Snail mRNA homeostasis during HCC progression [13]. HBXIP triggered metabolism reprogramming of HCC cells through METTL3-dependent m^6^A modified HIF-1*α* [14]. The hepatic microenvironment facilitated HCC proliferation and metastases through METTL3-mediated m^6^A modification of YAP1 [15]. YTHDF2 triggered HCC stem cell phenotype as well as metastases through modulating OCT4 expression via an m^6^A modification manner [16]. YTHDF2 deletion fueled inflammation as well as vascular abnormalization in HCC [17]. YTHDF2 weakened cellular proliferation and growth through destabilization of EGFR mRNA in HCC [18]. Nevertheless, to date, no experimental evidences have confirmed the biological significance of ZC3H13 in HCC pathogenesis.

Herein, we observed the biological roles of m^6^A regulator ZC3H13 in HCC as well as addressed the underlying mechanisms. Our data suggested that ZC3H13 suppressed the progression of HCC with m^6^A-PKM2-mediated glycolysis and sensitized HCC cells to cisplatin. Thus, our findings highlighted the critical functions of ZC3H13-mediated m^6^A modification in HCC and provided a promising therapeutic regimen against HCC.

## 2. Materials and Methods

### 2.1. Patients and Specimens

Primary HCC as well as adjacent control tissue specimens from 30 patients in the People's Hospital of Changshou Chongqing were harvested for this study. The inclusion criteria were as follows: (i) patients with pathologic diagnosis of HCC and (ii) patients who received curative removal. Meanwhile, patients with distant metastases at diagnosis were excluded. Informed consent was acquired from each patient. This research was carried out in line with the guidelines of the Ethics Committee of the People's Hospital of Changshou Chongqing and approved following the ethical standards of World Medical Association Declaration of Helsinki.

### 2.2. Bioinformatics Analysis

The Gene Expression Profiling Interactive Analysis (GEPIA) web server [19] was adopted for determining the mRNA expression of ZC3H13 in HCC and normal tissues retrieved from The Cancer Genome Atlas (TCGA) and the Genotype‐Tissue Expression (GTEx) projects. Survival analysis of HCC patients with high and low expression of ZC3H13 was presented via the Kaplan–Meier plotter (https://kmplot.com/analysis/). Difference of overall survival between groups was estimated with log-rank test.

### 2.3. Real-Time Quantitative Polymerase-Chain Reaction (RT-qPCR)

Total RNA was extracted utilizing TRIzol reagent (Beyotime, China). In total, 500 ng RNA was reversed transcribed through HiScript II 1st-Strand cDNA Synthesis kits (Takara, Beijing, China). RT-qPCR was carried out via ChamQ Universal SYBR qPCR Master Mix (Takara, Beijing, China) as well as LightCycler 480 instrument. The sequences of primers included the following: ZC3H13: 5′-TCTGATAGCACATCCCGAAGA-3′ (forward) and 5′-CAGCCAGTTACGGCACTGT-3′ (reverse); PKM2: 5′- ATGTCGAAGCCCCATAGTGAA-3′ (forward) and 5′-TGGGTGGTGAATCAATGTCCA-3′ (reverse); and GAPDH: 5′- CTGGGCTACACTGAGCACC-3′ (forward) and 5′-AAGTGGTCGTTGAGGGCAATG-3′ (reverse). Data were quantified with a comparative Ct method (2^−ΔΔCt^).

### 2.4. Western Blotting

Tissue and cell specimens were lysed with RIPA buffer (Beyotime, China) plus protease inhibitor cocktail. Lysed protein was extracted, and protein concentrations were evaluated with BCA kits (Sigma, USA). Afterwards, the equal amount of protein was separated with 12% SDS-PAGE as well as transferred to polyvinylidene difluoride membrane (Millipore, USA). After being blocked, the membrane was incubated by primary antibodies targeting ZC3H13 (1/2000; ab70802; Abcam, USA), GLUT (1/1000; ab156876; Abcam, USA), LDHA (1/5000; ab52488; Abcam, USA), LDHB (1/2000; ab264358; Abcam, USA), PKM2 (1/1000; ab85555; Abcam, USA), and *β*-actin (1/5000; ab179467; Abcam, USA). Then, protein bands were incubated by secondary anti-mouse or anti-rabbit secondary antibody (1/5000; ab7063/ab7090; Abcam, USA). Protein band was visualized with ECL assay kits.

### 2.5. Cell Culture

Human normal liver cells L-O2 as well as human liver cancer cell lines HUH-7, Hep3B, HepG2, and SMMC-7721 were retrieved from ATCC (USA). All cells were grown in Dulbecco's Modified Eagle's Medium (DMEM) supplemented with 10% fetal bovine serum (FBS) as well as 1% streptomycin/penicillin. Cells were grown in a humidified environment of 5% CO_2_ at 37°C.

### 2.6. Transfection

Short interfering RNA (siRNA) against ZC3H13 as well as PKM2 was synthesized for specifically silencing ZC3H13 as well as PKM2 expressions in Hep3B and HUH-7 cells. HCC cells transfected with scrambled siRNAs acted as si‐NC. The full-length ZC3H13 cDNA was synthesized and then subcloned into the pcDNA3.1 vector to establish pcDNA-ZC3H13 overexpression (OE-ZC3H13) plasmid. All plasmids were retrieved from GenePharma (Shanghai, China). Transient transfection was carried out lasting two days via Lipofectamine 3000.

### 2.7. Cell Counting Kit-8 (CCK-8) Assay

Hep3B as well as HUH-7 cells were planted onto 96-well plates (3,000 cells/well). In line with the protocols of CCK-8 kits (Dojindo, Japan), 10 *μ*L CCK-8 solution that was diluted by 100 *μ*L DMEM replaced the previous DMEM at diverse hours (24, 48, 72, and 96 h). After being cultured protecting from light at 37°C lasting an extra two hours, viable cells were determined through absorbance at 490 nm wavelength.

### 2.8. Clone Formation Assays

Hep3B as well as HUH-7 cells were seeded onto 6-well plates (1 × 10^3^ cells/well). Following incubation in a 5% humidified CO_2_ environment at 37°C lasting 2 weeks, HCC cells were gently washed by PBS twice as well as fixed by 4% paraformaldehyde lasting half an hour. Afterwards, the cells were stained by crystal violet lasting 30 min. The colonies formed (>50 cells/colony) were treated by crystal violet.

### 2.9. TdT-Mediated dUTP Nick-End Labeling (TUNEL) Staining

Hep3B and HUH-7 cells were planted onto 12-well plates. HCC cells were fixed with 4% paraformaldehyde lasting 15 min at room temperature, rinsed by PBS, and incubated by 3% H_2_O_2_ in methanol lasting 10 min. Afterwards, HCC cells were treated by 0.1% Triton X-100 lasting 2 min on ice as well as incubated by 50 *μ*L TUNEL reaction mixture lasting 60 min at 37°C in the dark. Following being rinsed by PBS, nuclei were labeled with DAPI. Finally, images were captured with a fluorescence microscope (Olympus, Japan).

### 2.10. Transwell Assays

For migration assays, Hep3B and HUH-7 cells were seeded onto the upper chamber as well as DMEM plus 20% FBS was added to the lower chamber. For invasion assays, Hep3B and HUH-7 cells were planted onto the upper chamber containing a Matrigel‐coated membrane (BD, USA). Following 48 h incubation, nonmigrative or noninvasive cells were moved away through wiping the upper side of the membrane utilizing sterile cotton bud; meanwhile, the migrative or invasive cells on the lower level of the membrane were stained by 0.5% crystal violet. Finally, HCC cells were counted for 6 randomly selected fields of view utilizing an IX71 inverted microscope (×200).

### 2.11. Cell Viability Assay

Hep3B as well as HUH-7 cells were planted onto 96-well plates (3,000 cells/well). Following being incubated overnight, DMEM plus distinct doses of cisplatin (0, 1, 2, 4, 8, 16, 60, and 32 *μ*M) replaced the original medium lasting three days. Afterwards, viable cells were investigated through CCK-8 assays. The drug half-maximum inhibitory concentration (IC50) values were finally determined.

### 2.12. Measurement of Glucose Uptake and Lactate Production

Glucose uptake as well as lactate production was separately tested through Glucose Uptake Colorimetric Assay Kits (BioVision, USA) and Lactate Colorimetric Assay Kits (BioVision, USA) in Hep3B as well as HUH-7 cells following the manufacturer's protocols.

### 2.13. mRNA Stability Assay

Stability of mRNA assays in Hep3B as well as HUH-7 cells was evaluated through incubating cells with 5 *μ*g/mL actinomycin D (Act-D, Sigma, USA). Afterwards, cells were harvested at the indicated time points, and mRNAs were drawn for RT-qPCR with GAPDH as the reference control.

### 2.14. RNA Immunoprecipitation (RIP) Assay

RIPA assay was conducted with Magna RIP RNA-Binding Protein Immunoprecipitation kits (Millipore, USA) in line with the manufacturer's instructions. Hep3B as well as HUH-7 cells were lysed utilizing RIPA lysis buffer. Cell lysate was immunoprecipitated through anti-ZC3H13 antibodies or nonimmunized IgG at 4°C overnight. Afterwards, RNA was purified and RT-qPCR was utilized for measuring the level of PKM2 transcript in ZC3H13 or IgG immunocomplex.

### 2.15. Luciferase Reporter Assay

Promoter sequence of PKM2 was cloned into pEZX-PL01 control vector containing firefly luciferase as well as Renilla luciferase. Luciferase assay was carried out utilizing Luc-Pair™ Duo-Luciferase HS Assay kits. In brief, pretreated Hep3B as well as HUH-7 cells were cotransfected by ZC3H13-wild-type (ZC3H13-WT) or ZC3H13-mutation-type (ZC3H13-MUT) as well as 250 ng pEZX-PL01 reporter plasmid (Promega, Shanghai, China) in 12-well plates. Following transfection lasting 6 h, HCC cells were seeded into 96-well plates. Following 36 h, cells were collected and analyzed utilizing Dual-Glo Luciferase Assay system. Activity of firefly luciferase was normalized to that of Renilla luciferase for evaluating the luciferase and transcriptional activity.

### 2.16. Methylated RNA Immunoprecipitation qPCR (MeRIP‐qPCR)

1 *μ*g·m^6^A and IgG antibodies were treated by Protein G Magnetic Beads in 1x reaction buffer at 4°C lasting 3 h as well as treated by 200 *μ*g isolated RNA at 4°C lasting 3 h. Bound RNAs were eluted via incubating by RNA-antibodies-conjugated bead plus 100 *μ*L Elution Buffer lasting 30 min at room temperature. Eluted RNAs were extracted through phenol: chloroform method in line with ethanol precipitation. Extracted m^6^A-RIP RNAs were reverse-transcribed as well as quantified via RT-qPCR. IPs enriched rates of transcripts were determined as the ratios of their amounts in IPs to those in the input generated from the same number of cells.

### 2.17. Statistical Analyses

Statistical analyses were conducted with GraphPad Prism 8 software (GraphPad Software, Inc., San Diego, CA, USA). Student's *t*-test and one- or two-way analysis of variance were utilized for comparisons between groups as appropriate. Kaplan–Meier method was conducted for measuring the survival curves, and differences were assessed with log-rank test. *P* values less than 0.05 were indicative of statistical significance.

## 3. Results

### 3.1. ZC3H13 Displays Low Expression in HCC and Correlates with Survival Outcomes

**Figure 1:**
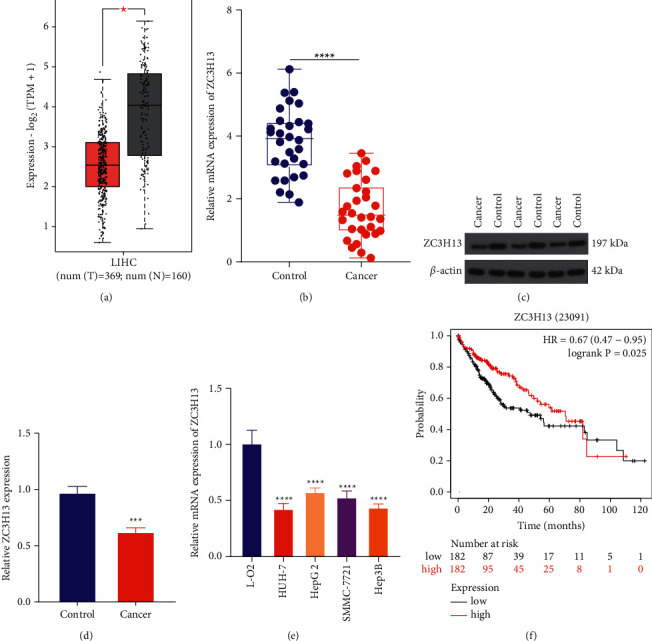
ZC3H13 displays low expression in HCC and correlates with survival outcomes. (a) Expression of ZC3H13 in 369 HCC and 160 control tissue specimens from TCGA and GTEx projects. (b) RT-qPCR examining the mRNA expression of ZC3H13 in 30 paired HCC as well as adjacent control tissue specimens in our cohort. (c, d) Western blotting detecting the protein expression of ZC3H13 in three HCC as well as adjacent control tissue specimens. (e) RT-qPCR testing the mRNA expressions of ZC3H13 in human normal liver cells L-O2 as well as human liver cancer cell lines HUH-7, Hep3B, HepG2, and SMMC-7721. (f) Kaplan–Meier survival analyses of HCC patients who possessed high and low expressions of ZC3H13 via the Kaplan–Meier plotter. ^*∗*^*P* < 0.05; ^*∗∗∗*^*P* < 0.001; and ^*∗∗∗∗*^*P* < 0.0001.

For investigating the underlying function of ZC3H13 in liver carcinogenesis, this study firstly tested the mRNA expressions of m^6^A methyltransferase ZC3H13 in 369 HCC tissues and 160 normal tissues from TCGA and GTEx projects. In Figure 1(a), ZC3H13 expressions were markedly downregulated in HCC relative to control tissues. Furthermore, similar mRNA expression patterns of ZC3H13 were verified in our cohort comprising of 30 paired cancerous and normal specimens (Figure 1(b)). Analysis of western blotting showed the decreased expression of ZC3H13 protein in HCC relative to normal tissues (Figures 1(c) and 1(d)). Moreover, our in vitro experiments confirmed the decreased mRNA expression of ZC3H13 in human liver cancer cell lines HUH-7, Hep3B, HepG2, and SMMC-7721 relative to human normal liver cells L-O2 (Figure 1(e)). Kaplan–Meier analysis uncovered that HCC patients with high ZC3H13 expression displayed a remarked survival advantage utilizing the online bioinformatics tool Kaplan–Meier plotter (Figure 1(f)). With the above evidences, ZC3H13 expression was remarkedly downregulated in HCC, which could be implicated in the pathogenesis and progression of HCC.

### 3.2. ZC3H13 Inhibits Cell Proliferation of HCC Cells

**Figure 2:**
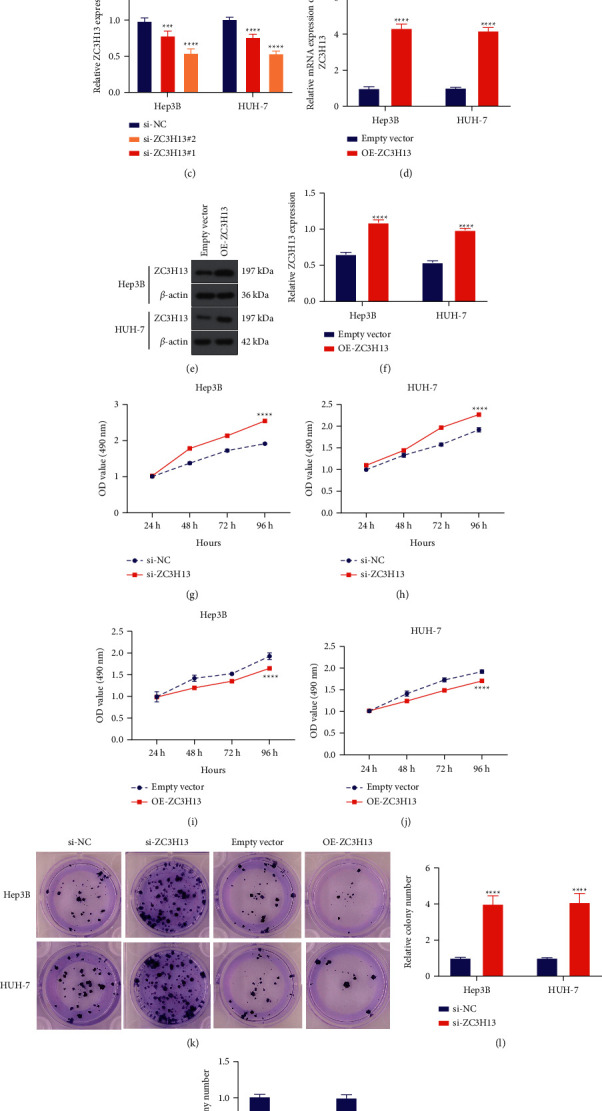
ZC3H13 alleviates cellular proliferative abilities of HCC cells. (a) RT-qPCR as well as (b, c) western blotting for detection of ZC3H13 expression in Hep3B and HUH-7 cells with siRNAs targeting ZC3H13. (d) RT-qPCR as well as (e, f) western blotting examining ZC3H13 expression for Hep3B as well as HUH-7 cells with ZC3H13 overexpression vectors. (g, h) CCK-8 examining the cellular growth for Hep3B as well as HUH-7 cells with ZC3H13 deficiency. (i, j) CCK-8 for evaluation for Hep3B as well as HUH-7 cells with ZC3H13 overexpression. (k–m) Clonogenicity assay for investigation of the colony formation for Hep3B as well as HUH-7 cells with ZC3H13 deficiency or overexpression. ^*∗∗∗*^*P* < 0.001; ^*∗∗∗∗*^*P* < 0.0001.

For addressing the effects of ZC3H13 on HCC progression, this study silenced ZC3H13 expression in Hep3B and HUH-7 cells (Figures 2(a)–2(c)), and its expression was upregulated in the two HCC cells (Figures 2(d)–2(f)) due to their relatively lower expression among all HCC cells, as determined with RT-qPCR and western blotting. CCK-8 results demonstrated that ZC3H13 deficiency enhanced cell growth in Hep3B as well as HUH-7 cells (Figures 2(g) and 2(h)). In contrast, cell growth of HCC cells was alleviated through overexpressed ZC3H13 (Figures 2(i) and 2(j)). As depicted in clonogenicity assay, clone formation of Hep3B and HUH-7 cells was remarkedly enhanced through ZC3H13 deficiency (Figures 2(k) and 2(l)). The opposite results were investigated when ZC3H13 was overexpressed (Figures 2(k) and 2(m)). Collectively, ZC3H13 might inhibit cell proliferation of HCC cells.

### 3.3. ZC3H13 Promotes Apoptosis and Suppresses Migration and Invasion in HCC Cells

**Figure 3:**
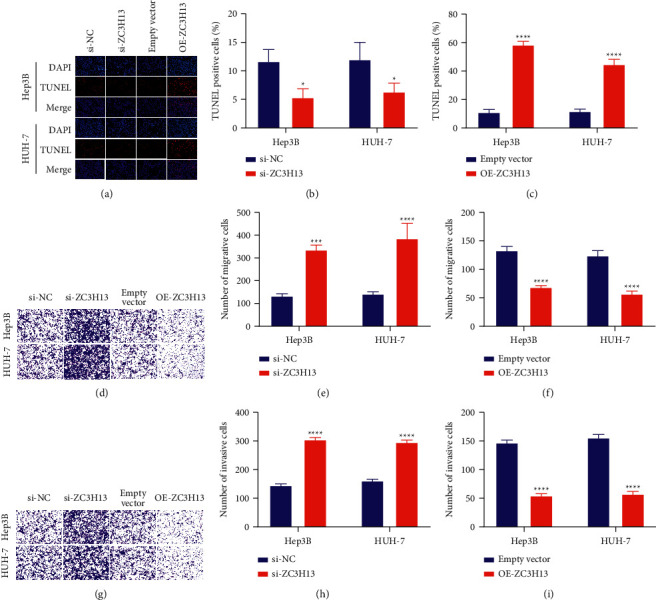
ZC3H13 promotes apoptosis and suppresses migration and invasion in HCC cells. (a–c) TUNEL for detecting the apoptotic levels for Hep3B as well as HUH-7 cells with ZC3H13 deficiency or overexpression. Magnification, 200×. (d–f) Evaluation of migration levels for Hep3B as well as HUH-7 cells with ZC3H13 deficiency or overexpression utilizing Transwell assays. Magnification, 200×. (g–i) Quantification of invasion levels for Hep3B as well as HUH-7 cells with ZC3H13 deficiency or overexpression utilizing Transwell assays. Magnification, 200×. ^*∗*^*P* < 0.05; ^*∗∗∗*^*P* < 0.001; and ^*∗∗∗∗*^*P* < 0.0001.

TUNEL assays were utilized for evaluating the effects of ZC3H13 on apoptosis of HCC cells. Our data showed that ZC3H13 deficiency reduced cell apoptosis, whereas ZC3H13 overexpression enhanced cellular apoptotic levels of Hep3B as well as HUH-7 cells (Figures 3(a)–3(c)). Transwell assays revealed that migrative capacities of Hep3B as well as HUH-7 cells were enhanced through ZC3H13 deficiency; meanwhile, overexpressed ZC3H13 alleviated the migrative capacities of HCC cells (Figures 3(d)–3(f)). We also noticed the increase in the invasive abilities of Hep3B and HUH-7 cells induced by ZC3H13 knockdown (Figures 3(g) and 3(h)). However, invasive abilities were reduced by ZC3H13 overexpression (Figures 3(g) and 3(i)). Taken together, ZC3H13 promoted apoptosis as well as suppressed migrative and invasive capacities of HCC cells.

### 3.4. ZC3H13 Increases Sensitivity to Cisplatin in HCC Cells

**Figure 4:**
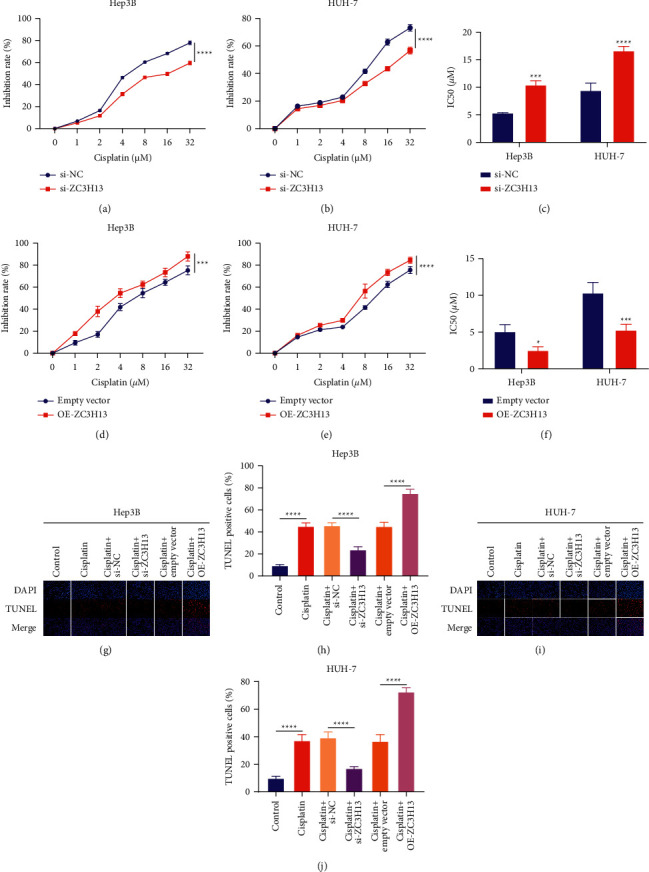
ZC3H13 increases sensitivity to cisplatin in HCC cells. (a, b) Inhibition rates of cisplatin for Hep3B as well as HUH-7 cells with ZC3H13 deficiency through CCK-8 assays. (c) Evaluation of IC50 values of cisplatin for Hep3B as well as HUH-7 cells with ZC3H13 deficiency. (d, e) Inhibition rates of cisplatin in Hep3B as well as HUH-7 cells with ZC3H13 overexpression by CCK-8 assays. (f) Assessment of IC50 values of cisplatin in Hep3B as well as HUH-7 cells with overexpressed ZC3H13. (g–j) TUNEL assays examining the apoptotic levels of Hep3B as well as HUH-7 cells with ZC3H13 deficiency or overexpression following exposure to cisplatin. Magnification, 200×. ^*∗*^*P* < 0.05; ^*∗∗∗*^*P* < 0.001; and ^*∗∗∗∗*^*P* < 0.0001.

We assessed the effects of ZC3H13 on sensitivity to cisplatin in HCC cells. Our CCK-8 data suggested that viable Hep3B as well as HUH-7 cells were suppressed as cisplatin was gradually increased (Figures 4(a) and 4(b)). ZC3H13 knockdown prominently reduced the inhibition rates of cisplatin in HCC cells relative to controls. Quantification analysis of IC50 values of cisplatin showed that ZC3H13 knockdown contributed to increased IC50 of cisplatin in Hep3B and HUH-7 cells, indicative of the reduced sensitivity to cisplatin (Figure 4(c)). Meanwhile, overexpressed ZC3H13 elicited the opposite effects (Figures 4(d)–4(f)). Moreover, our results uncovered that apoptotic levels of Hep3B as well as HUH-7 cells were markedly enhanced following treatment with cisplatin lasting 48 h (Figures 4(g)–4(j)). However, ZC3H13 knockdown weakened the inhibitory effects of cisplatin on apoptosis of HCC cells; meanwhile, overexpressed ZC3H13 enhanced the cisplatin-induced apoptotic levels. Above data demonstrated that ZC3H13 was capable of enhancing the cisplatin chemosensitivity of HCC cells.

### 3.5. ZC3H13 Reduces Metabolism Reprogramming of HCC Cells

**Figure 5:**
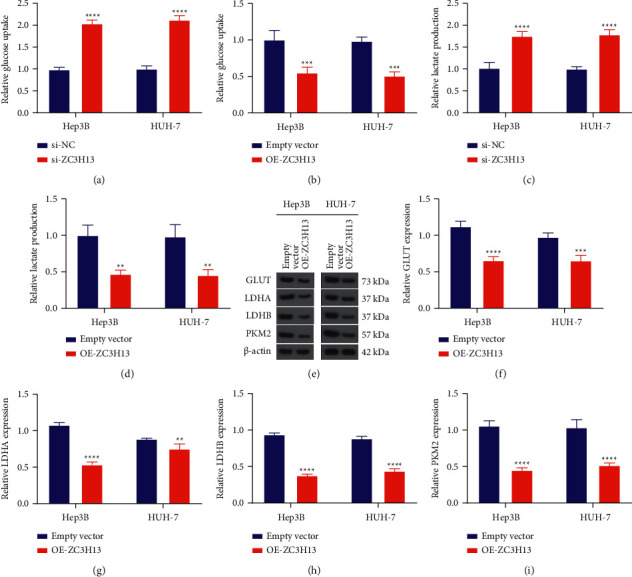
ZC3H13 weakens metabolism reprogramming of HCC cells. (a, b) Quantification of glucose uptake of Hep3B as well as HUH-7 cells with ZC3H13 deficiency and overexpression. (c, d) Quantification of lactate production of Hep3B as well as HUH-7 cells with ZC3H13 deficiency and overexpression. (e–i) Western blotting detecting the expression of metabolism reprogramming-related proteins including GLUT, LDHA, LDHB, and PKM2 in Hep3B as well as HUH-7 cells with overexpressed ZC3H13. ^*∗∗*^*P* < 0.01; ^*∗∗∗*^*P* < 0.001; and ^*∗∗∗∗*^*P* < 0.0001.

The Warburg effect represents a sign of metabolism reprogramming of cancer, in which most cancer cells exhibit enhanced glucose uptake as well as lactic acid production when there is sufficient oxygen supply [20]. Herein, we measured the effects of ZC3H13 on bioenergy metabolism levels of HCC cells. Our data demonstrated that ZC3H13 deficiency increased glucose uptake of Hep3B and HUH-7 cells, whereas ZC3H13 overexpression reduced glucose uptake (Figures 5(a) and 5(b)). Moreover, we noticed that lactate production was enhanced by ZC3H13 knockdown, and the opposite results were investigated when ZC3H13 was overexpressed (Figures 5(c) and 5(d)). Through western blotting, the expressions of glycolysis-related proteins GLUT, LDHA, LDHB, and PKM2 were measured in Hep3B and HUH-7 cells (Figure 5(e)). As a result, ZC3H13 overexpression remarkedly decreased the expressions of GLUT, LDHA, LDHB, and PKM2 proteins in HCC cells (Figures 5(f)–5(i)). In conclusion, ZC3H13 modulated metabolism reprogramming of HCC cells.

### 3.6. ZC3H13-Mediated m^6^A Modification Reduces PKM2 mRNA Stability

**Figure 6:**
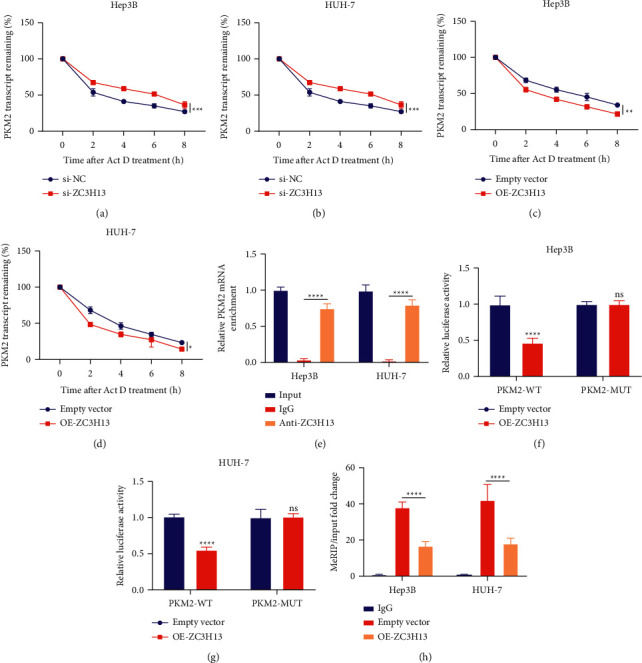
ZC3H13-mediated m^6^A modification reduces PKM2 mRNA stability in HCC cells. (a, b) Detection of the remaining PKM2 transcript in Hep3B as well as HUH-7 cells with ZC3H13 knockdown following exposure to Act D for the indicated time points. (c, d) Detection of the remaining PKM2 transcript for Hep3B as well as HUH-7 cells with ZC3H13 overexpression under exposure to Act D for the indicated time points. (e) RIP assay examining the enrichment levels of PKM2 mRNA in Hep3B as well as HUH-7 cells under incubation by anti-ZC3H13 or anti-IgG antibody. GAPDH transcript was utilized as a control. (f, g) Luciferase reporter assay examining the effects of ZC3H13 on wild-type PKM2 (PKM2-WT) or mutant PKM2 (PKM2-MUT) vector for Hep3B as well as HUH-7 cells. (h) MeRIP‐qPCR detecting m^6^A modification levels of PKM2 through immunoprecipitation of m^6^A-modified mRNA for Hep3B as well as HUH-7 cells with empty vector or ZC3H13 overexpression. Ns: not significant; ^*∗*^*P* < 0.05; ^*∗∗*^*P* < 0.01; ^*∗∗∗*^*P* < 0.001; and ^*∗∗∗∗*^*P* < 0.0001.

ZC3H13-silencing and overexpressing HCC cells were treated by Act D. Our results showed that ZC3H13 knockdown remarkedly increased the remaining PKM2 transcripts for Hep3B as well as HUH-7 cells (Figures 6(a) and 6(b)). Rather, ZC3H13 overexpression reduced the remaining PKM2 transcripts in two HCC cells (Figures 6(c) and 6(d)). The data indicated that ZC3H13 could decrease the stability of PKM2 mRNA. Moreover, RIP results showed that anti-ZC3H13 antibody prominently enriched the levels of PKM2 mRNA relative to anti-IgG antibody in Hep3B and HUH-7 cells (Figure 6(e)). However, GAPDH transcript was not detected in ZC3H13 or IgG immunocomplex. Thus, ZC3H13 possessed the capacity of binding to PKM2 transcript physically. Moreover, we further investigated whether PKM2 3′-untranslated region (3′-UTR) was required for ZC3H13 for reducing PKM2 expression. Therefore, dual-luciferase assay was carried out. Our data demonstrated that ZC3H13 overexpression remarkedly lowered the luciferase activities of PKM2 3′-UTR reporter vector for Hep3B as well as HUH-7 cells (Figures 6(f) and 6(g)). However, no effect was investigated for the empty vector. Thus, above data were indicated that ZC3H13 bound to PKM2 3′-UTR. In line with MeRIP‐qPCR results, ZC3H13 overexpression reduced the m^6^A levels of PKM2 mRNA for Hep3B as well as HUH-7 cells (Figure 6(h)). Thus, the findings indicated that ZC3H13 decreased the stability of PKM2 mRNA with an m^6^A-dependent manner.

### 3.7. PKM2 Knockdown Weakens Cell Proliferation and Metabolic Reprogramming Mediated by ZC3H13 in HCC Cells

**Figure 7:**
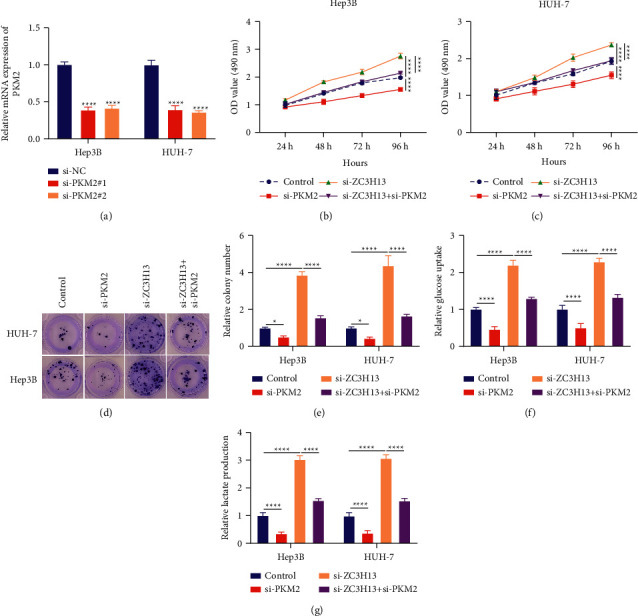
PKM2 knockdown weakens cell proliferation and metabolic reprogramming mediated by ZC3H13 in HCC cells. (a) RT-qPCR detecting the mRNA expression of PKM2 for Hep3B as well as HUH-7 cells transfected by siRNAs against PKM2. (b, c) CCK-8 examining the effects of PKM2 knockdown on cell growth for Hep3B as well as HUH-7 cells with ZC3H13 loss. (d, e) The effects of silencing or overexpressing PKM2 on clone formation of Hep3B as well as HUH-7 cells with ZC3H13 knockdown. (f, g) The effects of PKM2 knockdown on glucose uptake and lactate production in Hep3B and HUH-7 cells with ZC3H13 deficiency.^*∗*^*P* < 0.05; ^*∗∗∗∗*^*p* < 0.0001.

We further investigated the effects of interactions of ZC3H13 with PKM2 on HCC progression. We firstly confirmed the successful knockdown of PKM2 for Hep3B as well as HUH-7 cells with si-PKM2 transfections (Figure 7(a)). Afterwards, we assessed the cellular proliferation of PKM2 interacted with ZC3H13 in HCC through CCK-8. Our data demonstrated that PKM2 knockdown induced a prominent reduction in cell viability. Nevertheless, PKM2 knockdown reversed the cell growth mediated by ZC3H13 deficiency in Hep3B and HUH-7 cells (Figures 7(b) and 7(c)). Clone formation of HCC cells was weakened by PKM2 knockdown (Figures 7(d) and 7(e)). However, silencing ZC3H13 remarkedly ameliorated the clone formation induced by ZC3H13 knockdown in HCC cells. By quantitative analyses of glycolysis, we noticed that PKM2 deficiency prominently reduced glucose uptake and lactic acid production for Hep3B as well as HUH-7 cells (Figures 7(f) and 7(g)). But silencing PKM2 alleviated glucose uptake and lactic acid production induced by ZC3H13 deficiency. Taken together, ZC3H13 alleviated HCC cell proliferation by PKM2-dependent glycolytic signaling.

### 3.8. PKM2 Deficiency Alleviates Migration and Invasion Induced by ZC3H13 in HCC Cells

**Figure 8:**
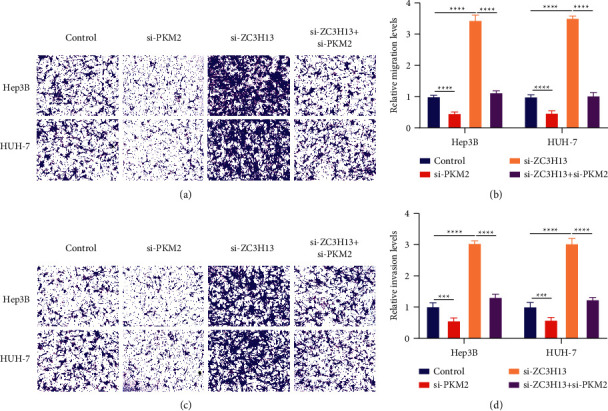
PKM2 deficiency alleviates migration and invasion induced by ZC3H13 in HCC cells. (a, b) Transwell assays detecting the effects of PKM2 knockdown on migrative capacities of Hep3B as well as HUH-7 cells with ZC3H13 deficiency. (c, d) Transwell assays detecting the effects of PKM2 knockdown on invasive capacities of Hep3B as well as HUH-7 cells with ZC3H13 deficiency. Magnification, 200×. ^*∗∗∗*^*p* < 0.001; ^*∗∗∗∗*^*p* < 0.0001.

The effects of interactions of ZC3H13 with PKM2 on HCC metastasis were investigated through quantification of migration and invasion via transwell assays. Our results demonstrated that PKM2 deficiency remarkedly alleviated the migrative capacities of Hep3B and HUH-7 cells (Figures 8(a) and 8(b)). Additionally, its deficiency reversed the migrative abilities induced by ZC3H13 knockdown in HCC cells. As depicted in Figures 8(c) and 8(d), silencing PKM2 led to a remarked decrease in the invasive abilities of Hep3B and HUH-7 cells. Also, PKM2 deficiency reversed the invasion of HCC cells mediated by ZC3H13 knockdown. Collectively, PKM2 deficiency alleviated migration and invasion induced by ZC3H13 in HCC cells.

## 4. Discussion

m^6^A modification of RNAs acts as a novel layer of epigenetic modulation [8]. The biochemical event exerts critical roles in modulating growth, differentiation, resistance, and metabolic reprogramming of cancer cells via modulation of RNA splicing, translation, and stability [14, 21, 22]. Several evidences have proposed m^6^A as a major modified type of mRNAs [23–25]. In our study, our evidences confirmed the important roles of m^6^A regulator ZC3H13 in HCC progression and uncovered the underlying mechanisms. Our results demonstrated that overexpressed ZC3H13 weakened malignant behaviors of HCC cells through m^6^A-PKM2-mediated glycolysis and enhanced chemosensitivity.

Consistent with bioinformatics analysis, ZC3H13 expression was downregulated in HCC as well as its loss correlated to dismal survival outcomes [26–28]. ZC3H13 weakens proliferative and invasive capacities of colorectal carcinoma cells through inactivating Ras-ERK pathway [29]. ZC3H13 is predictive of immune phenotype and therapeutic responses in renal carcinoma [30]. Our data demonstrated that overexpressed ZC3H13 alleviated proliferation, migration, and invasion as well as aggravated apoptosis in HCC cells, confirming that ZC3H13 exacerbated malignant behaviors of HCC cells. Tumor metastases and chemoresistance act as the major causes of therapeutic failure and increased mortality for HCC [31]. In line with the perspective of precision medicine, it is an urgency for finding novel molecular targets upon developing more effective therapeutic regimen. Herein, ZC3H13 overexpression could sensitize HCC cells to cisplatin, providing novel evidences for HCC chemotherapy.

HCC represents a heterogeneous malignancy, characterized by diverse etiological factors, that is implicated in metabolic alterations [32]. Previous evidences have demonstrated the significance of metabolic normalization to HCC inhibition [33–35]. The Warburg effect is fundamental to metabolic reprogramming in HCC progression [36]. Enhanced glucose uptake and lactate production maintain long‐term growth of cancer cells. Hopefully, reprogramming of HCC cells may manifest itself as a new insight into developing therapeutic regimen against HCC. Our data demonstrated that ZC3H13 had much potential of inhibiting glycolysis in HCC through modulating metabolism reprogramming. Our further analyses uncovered that ZC3H13-mediated m^6^A modification substantially alleviated PKM2 mRNA stability as well as overexpressed ZC3H13 facilitated malignant behaviors of HCC cells through PKM2-dependent glycolytic signaling. Even so, more selective and efficacious agents activating ZC3H13 will be developed upon HCC therapy in our future studies. There are several limitations in our study. Firstly, we collected 30 pairs of HCC specimens and matched nontumor specimens, and our results revealed that ZC3H13 expression was downregulated in HCC specimens. However, the sample size is small. The expression of ZC3H13 will be verified in larger HCC cohorts. Secondly, the biological function of ZC3H13 in HCC progression will be investigated through in vivo experiments.

## 5. Conclusion

In all, our evidences demonstrated that overexpressed ZC3H13 alleviated malignant behaviors and metabolism reprogramming of HCC cells through mediating the m^6^A-modified PKM2 mRNA. Therefore, ZC3H13 possessed the potential as a therapeutic target against HCC. Effective treatments for HCC might be conducted on the basis of the new molecular mechanisms proposed in these observations.

## Data Availability

The datasets analyzed during the current study are available from the corresponding author upon reasonable request.

## Abbreviations

HCC: Hepatocellular carcinoma
TACE: Transarterial chemoembolization
m^6^A: N^6^-Methyladenosine
GEPIA: Gene Expression Profiling Interactive Analysis
TCGA: The Cancer Genome Atlas
GTEx: Genotype‐tissue expression
RT-qPCR: Real-time quantitative polymerase-chain reaction
DMEM: Dulbecco's Modified Eagle's Medium
siRNAs: Short interfering RNAs
CCK-8: Cell counting kit-8
TUNEL: TdT-mediated dUTP nick-end labeling
IC50: Half-maximum inhibitory concentration
Act-D: Actinomycin D
RIP: RNA immunoprecipitation
MeRIP‐qPCR: Methylated RNA immunoprecipitation qPCR.
